# Investigating genetic diversity within *Cryptosporidium parvum* outbreaks using multi-locus variable number tandem repeat analysis

**DOI:** 10.1016/j.crpvbd.2025.100332

**Published:** 2025-10-31

**Authors:** Ross Bacchetti, Paula McCormack, Lisa Connelly, Derek J. Brown, Dominique L. Chaput, Claire L. Alexander

**Affiliations:** Scottish Microbiology Reference Laboratories (Glasgow), Glasgow Royal Infirmary, 10-16 Alexandra parade, Glasgow, G31 2ER, United Kingdom

**Keywords:** *Cryptosporidium parvum*, Outbreak investigation, MLVA, *gp60*, Subtyping

## Abstract

*Cryptosporidium parvum* is a zoonotic protozoan parasite of human and veterinary public health concern that causes gastrointestinal disease. Animal contact is a major risk factor for *C. parvum* outbreaks which require thorough investigation through the use of molecular subtyping. Recently, a multi-locus variable-number tandem repeat analysis (MLVA) scheme was established for *C. parvum*, offering improved subtyping resolution compared to the commonly used single-locus 60 kDa glycoprotein gene (*gp60*) subtyping approach. Using the *C. parvum* MLVA scheme, the genetic diversity of known *gp60* subtyped faecal DNA extracts collected between April 1st 2023 and March 31st 2024 was explored. A representative group of a common Scottish *gp60* subtype (IIaA15G2R1, *n* = 28) was analysed by MLVA and found to consist of 8 distinct complete MLVA profiles, with 4-12-5-7-27-36-16 (*n* = 12) being the most common. Genetic diversity within samples involved in three historic animal contact outbreaks (Outbreaks A, B and C) was investigated. Outbreak A, involving a single *gp60* subtype (IIaA19G1R1), consisted of only one MLVA profile (4-12-5-8-27-15-17). Outbreak B was caused by two *gp60* subtypes (IIaA17G1R1 and IIaA15G2R1), which were further subdivided into four MLVA profiles, two per *gp60* subtype (4-14-4-7-27-37-15 and 4-14-5-7-27-27-15, and 4-13-4-8-27-31-17 and 4-12-5-7-27-42-16, respectively). Lastly, Outbreak C, thought to have two-point sources of infection, involved one *gp60* subtype (IIaA15G2R1), which was subdivided into four distinct MLVA profiles (4-12-5-7-27-36-16, 4-12-5-7-27-32-15, 4-12-5-7-27-30-15, and 4-14-5-7-36-33-15). Improved MLVA resolution allowed outbreak specimens with insufficient epidemiological data to be linked to a source through sharing a common MLVA profile.

## Highlights

• A multi-locus approach investigating genetic diversity of *Cryptosporidium parvum gp60* subtypes.
• Eight complete MLVA profiles were identified from a group of common gp60 subtype IIaA15G2R1.
• Improved MLVA resolution enhanced outbreak investigation compared to *gp60* analysis.

## Graphical abstract

**Figure:**
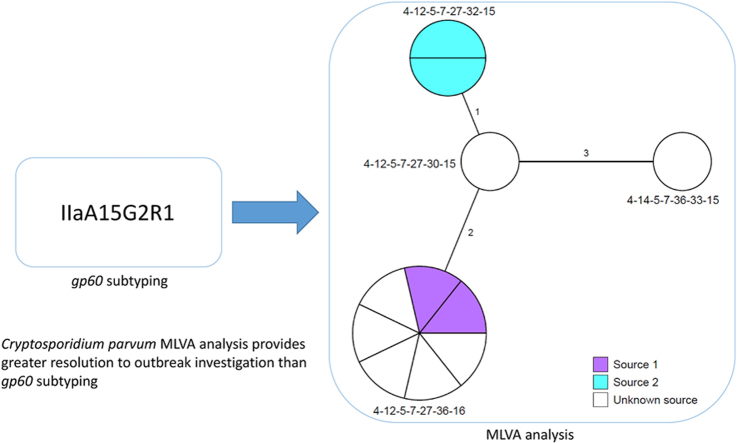

## 1 Introduction

The zoonotic protozoan parasite *Cryptosporidium parvum* is a well-known cause of gastrointestinal disease in animals, presenting as an organism of major concern for both human and veterinary public health (Xiao et al., 2004; Thomson et al., 2017; Ali et al., 2024). Clinical symptoms of human cryptosporidiosis can include diarrhoea, cramps, nausea, dehydration and fever (Farthing, 2000), with more severe disease occurring in young individuals and the immunocompromised (O’Connor et al., 2011; Bouzid et al., 2013; Kotloff et al., 2013; Lanternier et al., 2017). Transmission occurs faecal-orally; either from direct contact with human or animal faeces containing infectious oocysts, or through the consumption of food or water contaminated with oocysts (Koutsoumanis et al., 2018; Betancourt and Rose, 2004).

*Cryptosporidium parvum* infections can arise from various potential sources and reservoirs, including the environment, as well as farmed and wild animal populations (Ryan et al., 2014). Therefore, robust outbreak investigation tools are required to identify any human cases linked to a point source of infection. One frequently employed molecular subtyping method for outbreak investigation uses the 60 kDa glycoprotein gene (*gp60*) (Strong et al., 2000). This approach involves amplifying a region of the *gp60* gene, followed by Sanger sequencing to analyse hypervariable satellite regions, from which a subtype can be inferred. Over time, this single-locus approach has been widely adapted to permit subtyping of different *Cryptosporidium* species (Robinson et al., 2025), including those that cause the majority of human infection, *C. parvum* and *C. hominis* (Feng et al., 2018).

Recently, efforts were made to establish a multi-locus variable-number tandem repeat analysis (MLVA) scheme for *C. parvum* (Robinson et al., 2022). This approach aimed to provide greater discriminatory power through a fragment size-based method using seven variable number of tandem repeat (VNTR) markers, as opposed to the single-locus approach of *gp60* subtyping. Application of this method in 2022 to study seasonal *C. parvum* cases in Wales and the northwest of England highlighted the greater discriminatory power of this approach, and improved capability of identifying additional cases in known outbreaks, as well as an entirely new outbreak which would not have been identified otherwise (Risby et al., 2023).

In our study, we applied the *C. parvum* MLVA scheme to explore the genetic diversity within samples of known *gp60* subtypes. This is achieved through MLVA analysis of a representative group by selecting a common Scottish *gp60* subtype (IIaA15G2R1), and retrospective MLVA analysis of three outbreaks where *gp60* subtypes had previously been assigned.

## 2 Materials and methods

### 2.1 The Scottish *Cryptosporidium* Surveillance and Outbreak Service

The Scottish Microbiology Reference Laboratories (SMiRL), Glasgow, conduct speciation and molecular typing of confirmed *Cryptosporidium-*positive patient faecal samples as part of the Scottish Government-funded *Cryptosporidium* Surveillance and Outbreak Service. Positive faecal samples are first identified by NHS Diagnostic Microbiology Laboratories throughout Scotland, then sent to SMiRL for further molecular work-up.

Subtyping of a selection of positive samples originating from rural, semi-rural and urban locations using the *gp60* approach is performed routinely, to survey subtypes currently circulating in the Scottish population. Additionally, samples from suspected outbreak situations are also subjected to molecular subtyping. Data produced by SMiRL is shared with Public Health Scotland for further epidemiological investigation.

All data represented in this study are derived from *C. parvum-*positive human faecal samples received and processed by SMiRL between April 1st 2023 and March 31st 2024.

### 2.2 Faecal sample processing and DNA extraction

Faecal samples were transported to the SMiRL at room temperature. Oocysts present in faecal samples were concentrated using Mini Parasep® SF concentrators (Apacor, Berkshire, UK). This concentration step is standard practice within the laboratory, as this filters out larger pieces of debris within faecal samples which have the potential to interfere with the downstream DNA extraction process. Oocyst concentrates were produced by mixing a level scoop of solid faeces (scoop provided with kit, approximately 0.5 g solid faeces), or two-level scoops of liquid faeces in 3.3 ml of sterile molecular grade water. The faecal slurry was then vortexed for approximately 1 min, before being centrifuged at 1500 *rpm* for 2 min to filter faecal slurry through the Mini Parasep® SF inbuilt filter. Using a Pasteur pipette, the supernatant was carefully removed, leaving enough supernatant so that the collection cone of the Parasep® SF concentrator remains filled. The remaining supernatant within the collection cone was used to resuspend the concentrated pellet at the tip of the collection cone.

Resuspended oocyst concentrates underwent DNA extraction using an automated NucliSENS easyMAG platform (BioMerieux, Basingstoke, UK). Two hundred microlitres of each resuspended concentrate was loaded into easyMAG 8-well sample vessels. The eighth position of each sample vessel was reserved for an extraction control which consisted of 200 μl of sterile molecular-grade water. Sample vessels containing oocyst concentrates and extraction controls were loaded onto the easyMAG platform and extracted using the easyMAG Generic A run. Faecal DNA extracts were eluted in 100 μl of easyMAG elution buffer. Eluates were either stored at −20 °C or used immediately for real-time PCR.

### 2.3 Real-time PCR confirmation of *Cryptosporidium parvum-*positive samples

Faecal DNA extracts were tested for *C. parvum* by multiplex real-time PCR (RT-PCR), capable of detecting both *C. parvum* and *C. hominis* through targeting the 18S rRNA gene (Hadfield et al., 2011). Negative and positive control samples were also amplified, consisting of nuclease-free water and well-characterised *C. parvum* and *C. hominis* DNA, respectively. Extraction controls produced during easyMAG extractions were also amplified alongside their respective faecal DNA extracts. Amplification was performed using a Light Cycler® 480 (Roche, West Sussex, UK). Only *C. parvum-*positive samples were further processed.

### 2.4 Molecular subtyping by *gp60* analysis

Confirmed *C. parvum* positive DNA faecal extracts underwent nested-PCR targeting the *gp60* gene, as previously described (Deshpande et al., 2015). Amplicons of the correct size (800 bp) were confirmed by gel electrophoresis (1.4% agarose gel with 0.5 μg/ml ethidium bromide) and then subjected to bi-directional Sanger sequencing on the Applied Biosystems™ 3500xL Genetic Analyzer (Applied Biosystems, Warrington, UK) using the BigDye Terminator v3.1 Cycle Sequencing Kit (Thermo Fisher, Renfrew, UK). The quality of raw sequence traces was assessed using either CLC Main Workbench 8 software (Qiagen, Manchester, UK) or BioEdit software (Hall, 1999). Analysis of forward and reverse sequence traces was initially carried out using the NCBI BLASTn tool (https://blast.ncbi.nlm.nih.gov/Blast.cgi) to identify characteristic *gp60* repeating trinucleotide repeats (Xiao, 2010), until November 2024, where SMiRL verified the use of the online bioinformatics tool CryptoGenotyper (Yanta et al., 2021) for rapid identification of *Cryptosporidium gp60* subtypes from Sanger sequencing traces (https://usegalaxy.eu/?tool_id=toolshed.g2.bx.psu.edu%2Frepos%2Fnml%2Fcryptogenotyper%2FCryptoGenotyper%2F1.0%2Bgalaxy0&version=latest).

### 2.5 Molecular subtyping by multi-locus variable-number tandem repeat analysis (MLVA) scheme

Following previously described methods (Robinson et al., 2022), *C. parvum-*positive DNA faecal extracts were amplified using both a 3-plex and 4-plex PCR, targeting all seven VNTR target loci: cgd1_470_1429 (cgd1); cgd4_2350_796 (cgd4); cgd5_10_310 (MSF); cgd5_4490_2941 (cgd5); cgd6_4290_981 (cgd6); cgd8_ 4440_NC_506 (cgd8); and cgd8_4840_6355 (MM19). Both multiplex PCRs were carried out using a Type-it Microsatellite PCR Kit (Qiagen, Manchester, UK), on the Applied Biosystems™ ProFlex™ PCR System (Applied Biosystems, Warrington, UK).

Fragment sizing of amplicons was carried out as previously described (Risby et al., 2023; Robinson et al., 2022) by diluting amplicons 10× with Hi-Di™ Formamide (Life Technologies Limited, Paisley, UK) and adding to a mastermix consisting Hi-Di™ Formamide and GeneScan™ 600 LIZ™ dye Size Standard v2.0 (Thermo Fisher, Renfrew, UK). Fragment sizing of amplicons was completed using an Applied Biosystems™ 3500xL Genetic Analyzer (Applied Biosystems, Warrington, UK).

BioNumerics software, version 7.6 (Applied Maths, Sint-Martens-Latem, Belgium), was used to perform fragment sizing analysis. In collaboration with the *Cryptosporidium* Reference Unit, Swansea, UK, a SMiRL MLVA database was constructed and validated using supplied positive reference DNA samples of well-characterised MLVA profiles. Construction of the database was based upon previously published work by the *Cryptosporidium* Reference Unit (Robinson et al., 2022; Risby et al., 2023). Any MLVA alleles identified which were not already included within the SMiRL BioNumerics database were confirmed by bi-directional Sanger sequencing using previously described primer sets (Robinson et al., 2022). Analysis of VNTR Sanger sequencing was performed using either Benchling Biological Software (https://www.benchling.com/) or CLC Main Workbench 8 software (Qiagen, Manchester, UK). Following sequencing analysis, fragment analysis was repeated for a total of five times to create a new bin range within the BioNumerics MLVA database by calculating mean band size ± 0.7 bp (Robinson et al., 2022; Risby et al., 2023).

Any sample lacking an allelic profile at a particular locus, as a result of no peak being present during fragment analysis and no presence of the recognised VNTR sequence by Sanger sequencing (even upon repeat testing), that allele was termed null (Ø).

### 2.6 Data visualisation

Data visualisation (alluvial plot) was performed using RStudio version 4.1.0 (R Core Team, 2022), using the packages *ggplot2* (Wickham, 2016), *ggalluvial* (Brunson and Read, 2023), and *viridis* (Garnier et al., 2023). Minimum spanning tree construction was carried out following calculation of similarity coefficient using categorical mapping and UPGMA clustering using BioNumerics software, version 7.6 (Applied Maths, Sint-Martens-Latem, Belgium).

## 3 Results

### 3.1 MLVA diversity within common *gp60* subtype

Between April 1st 2023 and March 31st 2024, SMiRL confirmed a total of 216 *C. parvum-*positive faecal specimens, from which 28 different *gp60* subtypes were identified. The most common *C. parvum gp60* subtype identified from this sample group was IIaA15G2R1 (*n* = 80). To investigate genetic diversity within one given *gp60* subtype, MLVA analysis was performed on a representative number of positive IIaA15G2R1 faecal extracts (*n* = 28). Of the 28 samples analysed, 20 samples produced a complete MLVA profile. Eight of the 28 samples analysed produced profiles where one to three alleles could not be identified (even upon re-testing); therefore, these alleles were termed null (Ø). One of the eight samples which contained null alleles also appeared to have a mixed profile at locus cgd5.

**Fig. 1:**
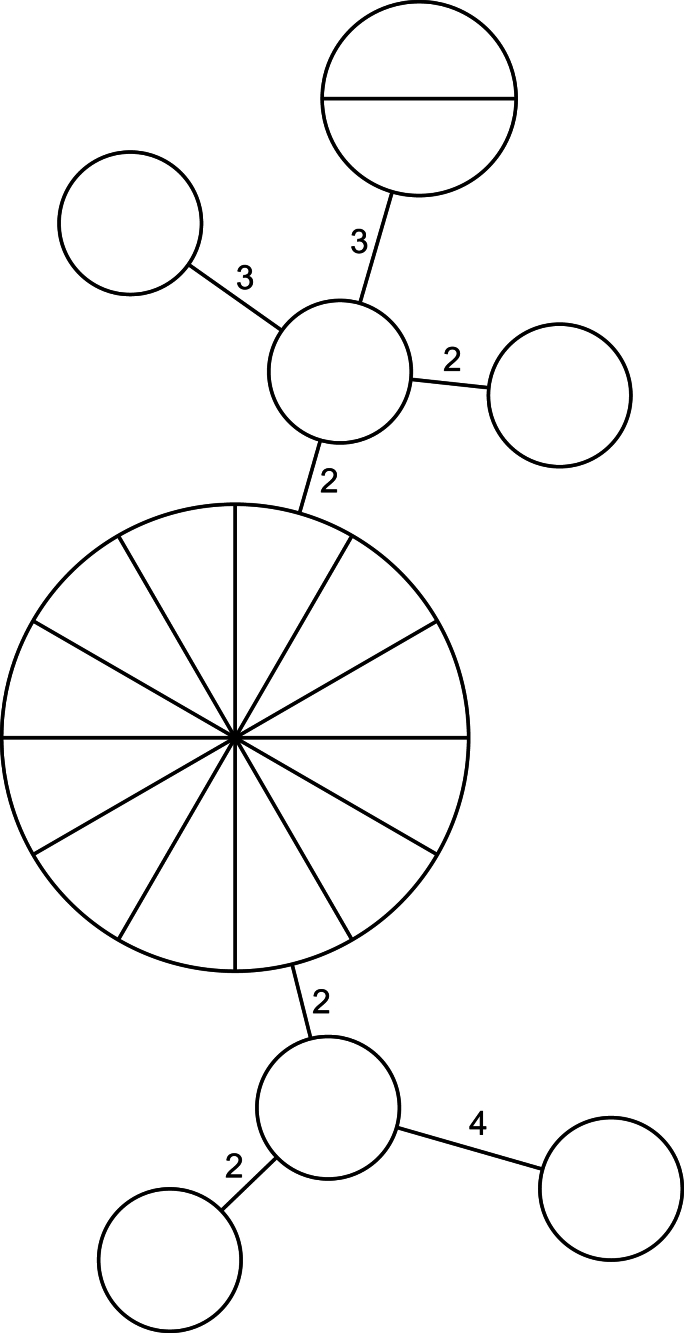
Minimum spanning tree showing the diversity of complete MLVA profiles extracted from specimens all subtyped as the common *gp60* subtype IIaA15G2R1. The number of allelic differences is shown between neighbouring clusters.

Eight distinct MLVA subtypes were identified from the 20 samples producing complete profiles (excluding those with null alleles). The most common MLVA profile detected was 4-12-5-7-27-36-16 (*n* = 12), followed by 4-13-5-8-27-28-15 (*n* = 2). The remaining MLVA profiles identified were detected only once (Supplementary Table S1). A minimum spanning tree was constructed using the 20 samples, which produced full MLVA profiles (excluding profiles with null alleles (Ø)), where each distinct MLVA profile identified was allocated a unique node (Fig. 1). Node size increased with the number of identical MLVA profiles within each node and allelic differences between neighbouring nodes were numerically represented upon each branch. The minimum spanning tree highlighted allelic diversity within this representative group of IIaA15G2R1 samples, albeit at a low level, with allelic differences between neighbouring clusters ranging from two to four (Fig. 1).

### 3.2 Benefit of MLVA resolution in outbreak investigation

To assess the genetic diversity highlighted by the MLVA scheme compared to *gp60* subtyping in outbreak settings, DNA extracted from faecal samples associated with three historic *C. parvum* animal contact outbreaks (Outbreaks A, B and C) was subtyped using both methods. All three outbreaks occurred between April 1st 2023 and March 31st 2024, within different geographical areas from one another, suspected to be the results of attending open farm events.

**Fig. 2:**
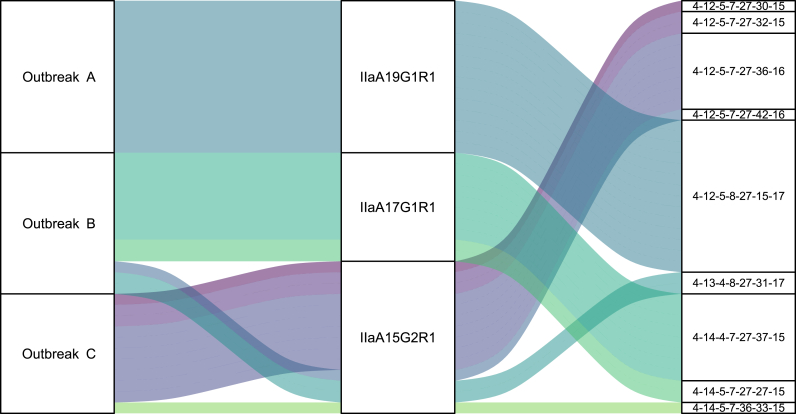
Alluvial plot highlighting associated *gp60* subtypes and MLVA profiles identified from specimens involved in each of the three outbreaks investigated.

Outbreak A, which occurred in April 2024 in the West of Scotland, involved a total of 14 samples, all of which were identified as *gp60* subtype IIaA19G1R1. Upon MLVA analysis, all 14 samples subtyped to a single profile: 4-12-5-8-27-15-17 (Fig. 2).

A total of 13 samples were associated with Outbreak B, which occurred in May/June 2023 in the West of Scotland. Two *gp60* subtypes were identified from this group of 13; IIaA17G1R1 (*n* = 10) and IIaA15G2R1 (*n* = 3). From conducting MLVA analysis on the same samples, four distinct MLVA profiles were obtained, two from each of the identified *gp60* subtypes. From IIaA17G1R1, 4-14-4-7-27-37-15 (*n* = 8) and 4-14-5-7-27-27-15 (*n* = 2) were identified, which differed from one another by two allelic differences. From IIaA15G2R1, 4-13-4-8-27-31-17 (*n* = 2) and 4-12-5-7-27-42-16 (*n* = 1) were identified, differing from one another by five allelic differences (Fig. 2).

Outbreak C, which occurred in April 2023, concerned a national increase of IIaA15G2R1 cases across Scotland. Eleven IIaA15G2R1-positive samples in particular, collected at the time of this outbreak, were suspected to be involved; however, a lack of epidemiological data made it difficult to link these cases to a potential source. At least two separate point sources were suspected to be the cause of these 11 positive samples; an open farm in the North of Scotland (termed as ‘Source 1’) and an open farm in the South of Scotland (termed as ‘Source 2’). Epidemiological data provided from Public Health Scotland (PHS) suggested that at least two of the eleven IIaA15G2R1 samples were suspected to be linked to Source 1, and another two of the eleven samples were linked to Source 2. The remaining seven samples did not have sufficient epidemiological data to suggest a link to either of the two potential sources. Analysis by MLVA identified four distinct MLVA profiles from these IIaA15G2R1 samples; 4-12-5-7-27-36-16 (*n* = 7), 4-12-5-7-27-32-15 (*n* = 2), 4-12-5-7-27-30-15 (*n* = 1), and 4-14-5-7-36-33-15 (*n* = 1) (Fig. 2). Three of the MLVA profiles identified (4-12-5-7-27-30-15; 4-12-5-7-27-32-15; and 4-12-5-7-27-36-16), all differed from one another by one to two allelic differences, whereas the fourth identified MLVA profile (4-14-5-7-36-33-15) differed from the other three profiles by three to four allelic differences. Epidemiological data confirmed that two 4-12-5-7-27-36-16 samples were linked to Source 1, and that the two 4-12-5-7-27-32-15 samples were linked to Source 2 (Table 1). Five of the specimens with an unknown source of infection shared the same MLVA profile as specimens linked to Source 1.

**Table 1:** Identified *gp60* subtypes and MLVA profiles of Outbreak C, highlighting MLVA can reveal genotypic linkage between specimens of an unknown infection source to specimens with a known source.

*gp60* subtype	MLVA profile	Source
IIaA15G2R1	4-12-5-7-27-30-15	Unknown
IIaA15G2R1	4-12-5-7-27-32-15	Source 2
IIaA15G2R1	4-12-5-7-27-32-15	Source 2
IIaA15G2R1	4-12-5-7-27-36-16	Unknown
IIaA15G2R1	4-12-5-7-27-36-16	Source 1
IIaA15G2R1	4-12-5-7-27-36-16	Source 1
IIaA15G2R1	4-12-5-7-27-36-16	Unknown
IIaA15G2R1	4-12-5-7-27-36-16	Unknown
IIaA15G2R1	4-12-5-7-27-36-16	Unknown
IIaA15G2R1	4-12-5-7-27-36-16	Unknown
IIaA15G2R1	4-14-5-7-36-33-15	Unknown

## 4 Discussion

In this study, we examined genetic diversity using the *C. parvum* multi-locus subtyping method (MLVA) within samples subtyped by the widely used single-locus *gp60* subtyping approach. Genetic diversity was first investigated within one common Scottish *gp60* subtype, IIaA15G2R1. Infection with this *gp60* subtype has previously been associated with animal contact and contaminated food products (McKerr et al., 2015; Chalmers et al., 2019), and it has remained one of the most common *gp60* subtypes within the UK in recent years (Deshpande et al., 2015; Chalmers et al., 2019; Bacchetti et al., 2023). MLVA analysis of these IIaA15G2R1 samples highlighted the greater discriminatory power of this multi-locus approach, subdividing this one *gp60* subtype into 8 separate complete MLVA profiles. This additional discriminatory power achieved with the MLVA scheme can enhance outbreak investigation and surveillance, allowing for further identification of genetically similar clusters within a single group of outbreak samples, which may have otherwise been allocated a single *gp60* subtype.

Of all the MLVA profiles identified from the IIaA15G2R1 samples, MLVA profile 4-12-5-7-27-36-16 appeared most frequently (*n* = 12). This finding may suggest that profile 4-12-5-7-27-36-16 is amongst those commonly affecting the Scottish population; however, this conclusion should not be drawn from this study alone. A previous study that genetically characterised *C. parvum* in dairy cows and calves during early calving season on a Central Scotland farm found that the IIaA15G2R1 *gp60* subtype was the most predominant subtype to infect adult cattle, and the only *gp60* subtype identified in calves (Bartley et al., 2023). However, the only complete MLVA profile obtained from IIaA15G2R1 samples (4-14-5-8-18-37-16) did not match any of the MLVA profiles obtained in our study, with the closest match to any of the profiles identified in our study differing at two alleles (4-14-5-8-18-27-17). To date, no other publication from a UK-based laboratory has reported the identification of the 4-12-5-7-27-36-16 profile, suggesting that this profile may be more commonly found in Scotland.

If the 4-12-5-7-27-36-16 MLVA profile identified in our study is indeed one of the most predominant throughout Scotland, then resolution of the MLVA scheme may not be able to differentiate all transmission chains, and a greater reliance on epidemiological data, such as known contact with risk at a given time and location, will be required to accurately link cases. This particular limitation of the MLVA scheme can be overcome using multi-locus sequence typing (MLST) which includes more VNTR markers than the seven used in the MLVA scheme (Troell et al., 2025) or employing a whole genome sequencing approach (Bayona-Vásquez et al., 2024). However, establishing the use of these higher discriminatory techniques requires time and additional funds to support an extension to SMiRLV’s usual scope of practice. The Scottish Microbiology Reference Laboratories will continue to monitor recurrently identified MLVA profiles over the coming years, to accurately document those commonly causing infection throughout the Scottish population.

Eight of the 28 IIaA15G2R1 DNA extracts, which underwent MLVA analysis, produced incomplete MLVA profiles, defined as containing a null (Ø) record for one or more alleles. Alleles could not be assigned to these null records following repeat analysis and Sanger sequencing of the loci in question. Previously published literature states that the presence of null alleles may be the result of poor DNA template, polymorphisms within the primer binding region, or indeed an absence of the allele (Risby et al., 2023). With these reasons in mind, it is important to consider that some of the null-containing profiles may indeed be more closely related to other fully identified profiles, particularly if they share the same value at five or six alleles. The MLVA scheme used in this study is limited to seven different loci, so the presence of a null allele detracts from the overall usefulness of that sample’s identified MLVA profile. Use of techniques providing greater discriminatory power, such as whole genome sequencing (Bayona-Vásquez et al., 2024) or an MLST scheme incorporating more VNTR markers (Troell et al., 2025), may provide data with greater epidemiological weight. Furthermore, the use of whole genome sequencing would aid in alleviating any dubiety around whether a null profile produced was the result of a polymorphism at the MLVA primer binding site, or the lack of an allele altogether.

Regarding the genetic diversity of known *gp60* profiles from three historic outbreaks occurring in three distinct geographical locations, Outbreak A was the result of *gp60* subtype IIaA19G1R1. This *gp60* subtype is a rarer subtype in the UK, but is well documented in outbreaks involving animal contact, particularly cattle (Lange et al., 2014; Utsi et al., 2016; Chalmers et al., 2019; de Alba et al., 2023). Only one MLVA profile was identified in these samples, 4-12-5-8-27-15-17, suggesting that parasites involved in this outbreak were genetically very similar, and that the point source of infection was genetically homogeneous.

In contrast, Outbreak B included two genetically diverse *gp60* subtypes: IIaA17G1R1, a common subtype identified in the UK in recent years, previously associated with cattle reservoirs and contact (Chalmers et al., 2019; Kaupke and Rzeżutka, 2022; Bacchetti et al., 2023; Hoque et al., 2023); and IIaA15G2R1, which as mentioned above, is a common UK *gp60* subtype, known to be responsible for animal contact and food-borne associated outbreaks. Individuals and groups of animals are capable of harbouring multiple species and multiple subtypes of *Cryptosporidium* at one time, which is likely to account for the genetic variation seen in Outbreak B (Bordes et al., 2020; Shaw et al., 2021). Conducting MLVA analysis on these *gp60* subtypes identified four MLVA subtypes, two for each *gp60* subtype. Both 4-14-4-7-27-37-15 and 4-14-5-7-27-27-15 were identified from IIaA17G1R1 samples, which shared more allelic similarity to one another, than 4-13-4-8-27-31-17 and 4-12-5-7-27-42-16 which were identified from the IIaA15G2R1 samples. This may suggest that *gp60* IIaA17G1R1 subtypes are more genetically conserved than IIaA15G2R1 subtypes; however, this would need to be further investigated given the small sample numbers involved in this study. It is possible that the four different MLVA profiles identified in Outbreak B were the result of preferential PCR amplification of different alleles present within the one faecal sample. It is also possible that the four MLVA profiles detected arose from genetic recombination, which can complicate epidemiological investigation by adding complexity when attempting to identify the true MLVA profile(s) responsible for cases. Both of these possibilities could explain the detection of multiple MLVA profiles within this sample group and should be considered when interpreting these data, but it would be difficult to determine whether one or both have occurred.

Unlike in the previous two outbreaks described, Outbreak C concerned a national increase in cases of a single common *gp60* subtype, IIaA15G2R1, in April 2023. Eleven positive IIaA15G2R1 samples collected at the time of this outbreak were suspected part of the outbreak, with limited epidemiological data suggesting at least two-point sources (two geographically distinct open farms) were involved. Different MLVA profiles were identified: 4-12-5-7-27-36-16, 4-12-5-7-27-32-15, 4-12-5-7-27-30-15, and 4-14-5-7-36-33-15. This suggests that *gp60* subtype IIaA15G2R1 may be genetically less conserved, as highlighted by the degree of allelic variation observed between identified MLVA profiles. Furthermore, this greater allelic diversity could be explained by the fact that two-point sources were assumed involved with this outbreak, both of which may have genetically distinct subtypes of *C. parvum*. Again, the limited sample size used in this study cannot definitively confirm this observation, and therefore, the genetic diversity within *gp60* subtype IIaA15G2R1 will continue to be monitored going forward. Considering epidemiological information, the two samples linked to Source 1 had MLVA profiles of 4-12-5-7-27-36-16. Five of the seven samples with insufficient epidemiological data to link them to one of the two potential sources were also identified to have the MLVA profile 4-12-5-7-27-36-16. This finding highlights the improved discriminatory power of the MLVA scheme over *gp60* subtyping, allowing molecular linkage of five of the unknown source samples to two samples known to be involved with Source 1 (Table 1). This ability to use the MLVA scheme to link specimens with outbreak cases and sources, where previously it may not have been possible due to limitations in subtyping tools available, supports another study (Risby et al., 2023). The MLVA profile identified for the two samples linked to Source 2 was 4-12-5-7-27-32-15. No other samples unlinked to sources shared this MLVA profile; however, one unknown source sample was identified to have a MLVA profile of 4-12-5-7-27-30-15, differing from the identified Source 2 MLVA profile by only one allelic difference. This suggests that the 4-12-5-7-27-30-15 sample is more likely linked to Source 2, possessing only one allelic difference from the Source 2 MLVA profile, as opposed to the two allelic differences from the Source 1 MLVA profile. The last sample unlinked to a source was found to have a MLVA profile of 4-14-5-7-36-33-15, which differed from all other MLVA profiles by three to four allelic differences. This suggests that perhaps the genetic diversity from one of the sources is very broad, or it may also suggest that perhaps this sample is not related to this outbreak and has been incorrectly assumed linked due to sharing a common *gp60* subtype.

When comparing all MLVA data produced in this study with previously published MLVA data, some common profiles were identified. First, profile 4-12-5-7-27-29-15 obtained from a IIaA15G2R1 sample in our study was identified by Robinson et al. (2022) from a IIaA17G1R1 sample identified from a group of ovine and bovine samples collected between 2001 and 2002 from North-West England and Wales. Likewise, profile 4-12-5-7-18-26-16 identified in our studied from a IIaA15G2R1 sample was also identified by Robinson et al. (2022) from a sample of the same *gp60* from the 2001–2002 ovine and bovine sample group. Profile 4-14-5-7-27-27-15, which we identified from two IIaA17G1R1 samples involved in Outbreak B, was also identified by Robinson et al. (2022) from a group of sporadic *C. parvum* cases from England and Wales in 2016. These findings suggest that these profiles have a wide geographical distribution and may have been circulating in the UK for many years. Profile 4-14-5-7-27-30-15, which we identified from a IIaA15G2R1 sample, was also identified by Risby et al. (2023), in two human samples from North-West England and one human sample from Wales collected between March 28th^,^ 2022 and July 31st^,^ 2022. This again suggests that profile 4-14-5-7-27-30-15 is geographically distributed across the UK.

## 5 Conclusions

This work highlights that the MLVA scheme provides greater discriminatory power when conducting molecular subtyping on *C. parvum* compared with the widely used single-locus approach of *gp60* subtyping. The MLVA scheme highlighted that the common UK *gp60* subtype IIaA15G2R1 consists of multiple distinct MLVA profiles, suggesting this one common subtype exhibits greater genetic diversity than suggested by *gp60* analysis alone. Lastly, using MLVA to investigate genetic diversity within historic *gp60* subtyped outbreak samples emphasised the benefit of using a tool with greater resolution. This was demonstrated through the identification of greater genetic diversity within outbreaks settings, providing further molecular evidence linking cases to potential sources of infection. To improve surveillance and outbreak investigations, SMiRL aims to integrate the *C. parvum* MLVA scheme into routine service to provide greater discriminatory power than the current *gp60* subtyping method.

## Ethical approval

Ethical approval was not required for this study, in line with local legislation and institutional requirements. Data were anonymised to protect patient confidentiality, and are stored securely within SMiRL as per the local National Health Services (NHS) Greater Glasgow and Clyde’s policies and procedures. In addition, the study forms part of a local Quality Management initiative to support improved service delivery.

## CRediT authorship contribution statement

**Ross Bacchetti:** Conceptualisation, Data curation, Formal analysis, Methodology, Project administration, Supervision, Validation, Visualisation, Writing - original draft, Writing - review & editing. **Paula McCormack:** Data curation, Formal analysis, Investigation, Validation, Writing - original draft. **Lisa Connelly:** Conceptualisation, Data curation, Formal analysis, Methodology, Supervision, Writing - review & editing. **Derek J. Brown:** Methodology, Resources, Software, Validation, Writing - review & editing. **Dominique L. Chaput:** Software, Visualisation, Writing - review & editing. **Claire L. Alexander:** Conceptualisation, Project administration, Supervision, Writing - review & editing.

## Funding

This work was carried out as part of the NHS National *Cryptosporidium* Outbreak and Surveillance Service. No external fund or grants were used for this study.

## Declaration of competing interests

The authors declare that they have no known competing financial interests or personal relationships that could have appeared to influence the work reported in this paper. Given their role as Guest Editor, Ross Bacchetti had no involvement in the peer review of this article and has no access to information regarding its peer review. Full responsibility for the editorial process for this article was delegated to Dr Frank Katzer (Co-Editor) and Professor Aneta Kostadinova (Editor-in-Chief).

## Data Availability

The data supporting the conclusions of this article are included within the article and its supplementary file.

## Appendix A Supplementary data

Supplementary Table S1 Details and results of all samples included in this study. Supplementary Table S1
